# Hepatic Inflammatory Response to Exogenous LPS Challenge is Exacerbated in Broilers with Fatty Liver Disease

**DOI:** 10.3390/ani10030514

**Published:** 2020-03-19

**Authors:** Wenqing Mei, Yarong Hao, Huilin Xie, Yingdong Ni, Ruqian Zhao

**Affiliations:** Key Laboratory of Animal Physiology & Biochemistry, Nanjing Agricultural University, Nanjing 210095, China; wenqingmei0523@163.com (W.M.); haoyarong01@163.com (Y.H.); sanatesidaqin@163.com (H.X.); Zhao.ruqian@gmail.com (R.Z.)

**Keywords:** fatty liver, corticosterone, LPS, inflammation, broiler

## Abstract

**Simple Summary:**

Fatty liver disease is the most common liver abnormality, characterized by lipid accumulation within the hepatocytes. The disease results in a significant economic burden on the poultry industry. Microbial infection is recognized as a continuous challenge worldwide. The current study evaluated hepatic function and inflammatory response in broilers with fatty livers following acute lipopolysaccharide (LPS) challenge. Results indicate that broilers suffering from fatty liver disease are more susceptible to the negative effects of LPS, showing inflammatory response activation and more severe damage to the liver.

**Abstract:**

This study aimed to examine hepatic function and inflammatory response in broilers with fatty livers, following acute lipopolysaccharide (LPS) challenge. One-day-old Lihua yellow broilers were fed a basal diet. Broilers were divided into four groups: control (CON), corticosterone treatment (CORT), LPS treatment (LPS), and LPS and CORT treatment (LPS&CORT). Results show that CORT induced an increase in plasma and liver triglycerides (TGs), which were accompanied by severe hepatic steatosis. The LPS group showed hepatocyte necrosis with inflammatory cell infiltration. Total liver damage score in the LPS&CORT group was significantly higher than that in the LPS group (*p* < 0.05). Activity levels of plasma alanine aminotransferase (ALT) and aspartate aminotransferase (AST) were similar in the CON and CORT groups, but higher in the LPS group. Gene expression upregulation of the proinflammatory cytokines (NF-κB, IL-1β, IL-6, IFN-γ, and iNOS) was also noted in the LPS group (*p* < 0.05). In particular, LPS injection exacerbated the gene expression of these proinflammatory cytokines, even when accompanied by CORT injections (*p* < 0.05). In summary, our results indicate that broilers suffering from fatty liver disease are more susceptible to the negative effects of LPS, showing inflammatory response activation and more severe damages to the liver.

## 1. Introduction

Non-alcoholic fatty liver disease (NAFLD) is an emerging health problem that affects a considerable proportion of adults in developing countries [1]. NAFLD is a complex, consisting of a range of diseases including hepatocellular steatosis, steatohepatitis, fibrosis, and irreversible cirrhosis [2]. The condition is tightly related to obesity, metabolic diseases, and insulin resistance [3]. In chickens, the relatively basic lymphatic system and chylomicrons transport of fat through the portal vein enhance fat synthesis and deposition in the liver [4]. Moreover, the de novo lipogenesis pathway is mainly active in the liver, similar to humans. Such a feature increases the susceptibility to fatty liver disease [5]. Thus, fatty liver disease is a concern for chickens, and causes a significant economic burden on the poultry industry [6]. It has been demonstrated that excessive glucocorticoid (GC) exposure can cause metabolic syndrome, including fatty liver disease, in both mammals and birds [7]. The active form of GC in chickens is primarily corticosterone (CORT), an indicator of stress [8]. Moreover, it is well known that treatment with exogenous CORT can cause abnormal fat deposition in the liver [9,10].

Microbial infection is recognized as a continuous challenge in the poultry industry. In the intensive poultry production system, broilers are confined to a limited space in large numbers [11], which exacerbates the risk of microbial infection. Approximately 70% of the liver’s blood supply arrives through the portal vein. Among other functions, the portal vein delivers substances absorbed through the intestine. Because of this, the liver is also the first barrier against intestinal pathogenic microorganisms such as bacteria and bacterial byproducts [12]. Bacterial products, such as lipopolysaccharide (LPS), a main component of the cell wall of gram-negative bacteria, are identified by the hepatic immune system and cause hepatic inflammation [13]. Intraperitoneal LPS injection was shown to simulate exogenous infection and induce hepatic inflammation [14]. Mechanisms of LPS-induced inflammatory response have been extensively explored. Extracellular LPS can be recognized by the toll-like receptor 4 (TLR4), which leads to an activation of myeloid differentiation factor 88 (MyD88). The triggering of MyD88 pathways ultimately leads to the transcription factor nuclear factor-κB (NF-κB) [15,16] and the activation of inflammatory cascades. This cascade produces various proinflammatory cytokines, such as tumor necrosis factor α (TNF-α), interleukin (IL)-1, and IL-6 [17]. C-reactive protein (CRP) and serum amyloid A (SAA) are acute-phase proteins. They are upregulated in response to LPS-induced inflammatory cytokines, leading to the acute-phase reaction.

Inflammation and fatty liver disease appear to be tightly linked. It has been reported that inflammasome-mediated dysbiosis regulates progression of NAFLD and obesity, and results in exacerbation of hepatic steatosis in mice [18]. Similarly, small doses of LPS administered to Wistar rats has promoted the development of NAFLD [19]. In contrast, a study by Sharifnia et al. showed that NAFLD increased fatty acid damage and hepatic sensitivity to LPS in a human cell culture model [20]. However, hepatic sensitivity to acute infection in birds with fatty liver disease remains unknown.

In this study, we hypothesized that exposure to LPS could exacerbate inflammatory responses in birds suffering from fatty liver disease. The aim of this study was to verify this hypothesis, using a CORT-induced fatty liver model and LPS-induced acute inflammation, to further explore how fatty liver disease affects the inflammatory response to an acute stimulus such as LPS.

## 2. Materials and Methods

All animal procedures in this study were approved by the Nanjing Agricultural University Animal Care and Use Committee (Approval #2012CB124703). Sampling procedures were in accordance with the “Guidelines on Ethical Treatment of Experimental Animals” (2006) No. 398, formulated by the Ministry of Science and Technology of China.

### 2.1. Animals and Treatment

One-day-old Lihua yellow broilers were purchased and raised at the Yangzhou Poultry Research Institute. All chicks had free access to food and water and received continuous illumination. Environmental temperature was maintained at 35 to 37 °C for the first week, then gradually decreased by approximately 3 °C per week until reaching a final temperature of 21 °C. The relative humidity was controlled at 55–65%. Dietary composition is shown in Table 1. After four weeks, broilers were randomly divided into four groups (n = 8/group): control (CON), CORT treatment (CORT), LPS treatment (LPS), and LPS and CORT treatment (LPS&CORT). Birds in the CON and LPS groups received vehicle treatment through daily subcutaneous injection of solvent (15% ethanol). Birds in the CORT and LPS&CORT groups received, for one week, CORT treatment (4.0 mg/kg) through daily (9:00–10:30 and 16:00–17:30) subcutaneous injections [10]. Corticosterone (C2505, Sigma-Aldrich, USA) was dissolved in 15% ethanol, and LPS (L2880, Sigma-Aldrich, St. Louis, MO, USA) was dissolved in saline. The LPS was extracted from *Escherichia coli* serotype O55:B5 by phenol. Body weight was recorded weekly from 1 to 35 days of age. At 35 days of age, two hours before sample collection, broilers from the LPS and LPS&CORT groups were injected intraperitoneally with 0.5 mg/kg LPS. Broilers from all groups were weighed and killed by rapid decapitation, in accordance with the American Veterinary Medical Association (AVMA) Guidelines for the Euthanasia of Animals (2013 Edition). Subsequently, blood samples were collected, centrifuged at 3500 rmp for 10 min, and the plasma was aspirated and stored at −20 °C, pending analysis. Liver samples were collected, rapidly frozen in liquid nitrogen, and kept at −80 °C until assayed. Liver tissue slices were also placed in 4% paraformaldehyde. 

### 2.2. Histopathology

For histological analysis, liver specimens fixed in 4% formaldehyde-buffered solution were embedded in paraffin, then sectioned. Samples were rated based on the severity of damage. The degree of damages in liver specimens was assessed by the histopathologist using three parameters including hepatocytic vacuolation, hepatocyte necrosis, and the proliferation of interstitial substance. A five-point scale (0–4 equaling none, slight, mild, moderate, and severe, respectively) was used to assess these three parameters [21]. In each case, three tissue sections were examined and a numerical score was assigned.

### 2.3. Plasma Biochemical Indicators Measurement and Triglycerides Content in the Liver 

Plasma activity of alanine aminotransferase (ALT) and aspartate aminotransferase (AST), and the levels of triglycerides (TG), total cholesterol (TCHOL), high- and low-density lipoprotein cholesterol (HDL and LDL, respectively), and glucose (GLU) were analyzed using an automatic biochemical analyzer (7020, HITACHI, Tokyo, Japan), as previously described [22]. Hepatic triglycerides were measured using a tissue triglyceride assay kit (E1013; Applygen Technologies Inc., Beijing, China) as previously described [8].

### 2.4. Reverse Transcription and Quantitative Real-Time PCR

Total RNA was extracted from liver samples (30 mg) with TRIzol Reagent (15596026, Invitrogen, Carlsbad, CA, USA). RNA concentration was assessed by a NanoDrop ND-1000 spectrophotometer (Thermo Fisher Scientific, Waltham, MA, USA). Subsequently, 500 ng of total RNA were reverse transcribed into cDNA, using random hexamer primers (Promega, USA). The cDNA was diluted 20× (1:20, *v/v*) before performing quantitative Real-Time PCR (RT-qPCR) on Mx3000 P Real-Time PCR System (Stratagene, San Diego, CA, USA). All primers were synthesized by Generay Biotech (Shanghai, China) and their sequences are listed in Table 2. The 18S rRNA gene was selected as a reference to normalize the expression of the other genes. The fold-expressions of the genes were analyzed by the 2^−ΔΔCT^ method [23].

### 2.5. Statistical Analysis

Statistical analysis was performed using SPSS 20.0 for Windows (SPSS 20.0, SPSS, IBM, Armonk, NY, USA). Descriptive statistics were used to examine homogeneity of variances, and normal distribution was assessed by a Shapiro–Wilk test. Log_10_ transformation was performed before analysis if data distribution was not normal. Data involving the four groups were analyzed by one-way Analysis of Variance (ANOVA), and the General Linear Model (GLM) procedure was conducted to analyze body weight. Graphical presentations were prepared using GraphPad Prism 5.0. Results are presented as means ± standard error of mean (SEM). Statements of statistical significance were based on the probability value *p* < 0.05.

## 3. Results

### 3.1. Growth Performance 

**Figure 1:**
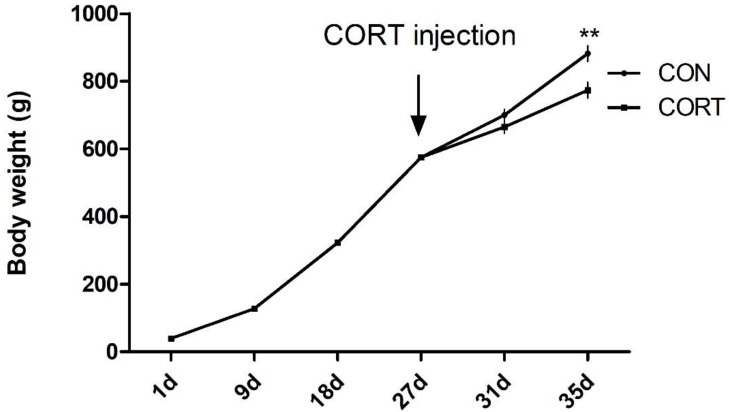
Changes in body weight. Data are presented as means ± SEM (n = 16); ** *p* < 0.01. CON, control group; CORT, corticosterone-treated group.

Broilers showed linear growth from 1 to 35 days of age. However, consecutive CORT injections for seven days significantly slowed weight gain when compared to control chickens (*p* < 0.01; Figure 1).

### 3.2. Hepatic Histological Analysis and Liver Damage Evaluation

**Figure 2:**
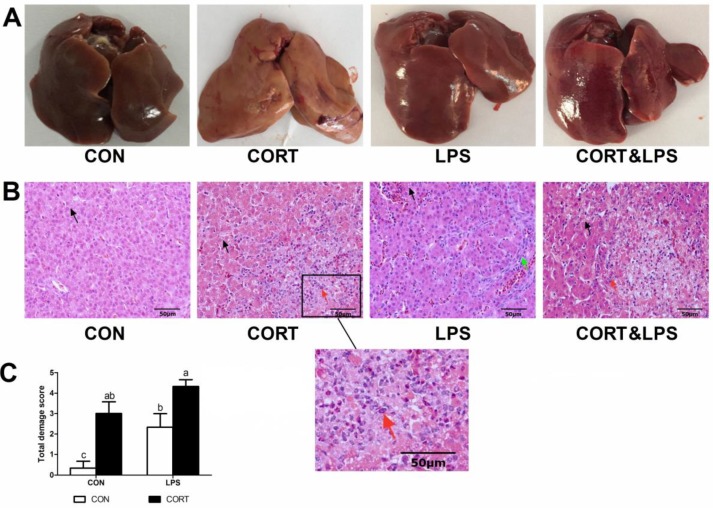
Phenotypic characterization of liver damages. (**A**) Phenotypic presentation of the liver. (**B**) Hepatic histological sections stained with hematoxylin and eosin (HE). (**C**) Total damage score of hepatic histological sections. Representative histological sections of the liver were observed with x40 magnification. Black, red, and green arrows indicate vacuolar degeneration, hepatocytes necrosis, and interstitial substance proliferation, respectively. CON, control group; CORT, corticosterone-treated group; LPS, lipopolysaccharide -treated group; CORT&LPS, corticosterone- and LPS-treated group. ^a–c^ Means with different small letter superscripts are significantly different (*p* < 0.05).

In the CON group, chicken livers exhibited normal size and red color, while CORT injection caused liver swelling as well as yellow coloration, which is indicative of steatosis (Figure 2A). LPS induced liver swelling and slight congestion, whereas injection of CORT, along with LPS, resulted in liver swelling and severe congestion (Figure 2A).

Hepatic histological sections stained with hematoxylin and eosin (HE) showed that fine droplets of fat were diffusely distributed through the hepatocytes of the CON group. However, more-extensive hepatocytic vacuolation due to fat deposition and hepatocyte swelling were observed in the CORT group. This manifestation was even more obvious in the CORT&LPS group. LPS challenge induced hepatic interstitial substance proliferation. Abnormalities such as dilated sinusoids and hepatocyte necrosis, accompanied by inflammatory cell infiltration, were also observed in the LPS groups (Figure 2B). As shown in Figure 2C, CORT or LPS injection alone significantly affected the total damage score (*p* < 0.05). However, the CORT&LPS combined treatment group showed a higher total damage score when compared to the LPS group (*p* < 0.05 Figure 2C).

### 3.3. Plasma Biochemical Indicators

As shown in Table 3, when compared with the CON group, CORT treatment significantly enhanced the levels of plasma TG, TCHOL, HDL, and LDL (*p* < 0.05), while LPS treatment decreased plasma TG concentration. The CORT&LPS group had lower plasma levels of TCHOL and HDL when compared to the CORT group (*p* < 0.05). There was no significant difference in plasma glucose concentration among these groups. 

### 3.4. Liver Damage Index 

**Figure 3:**
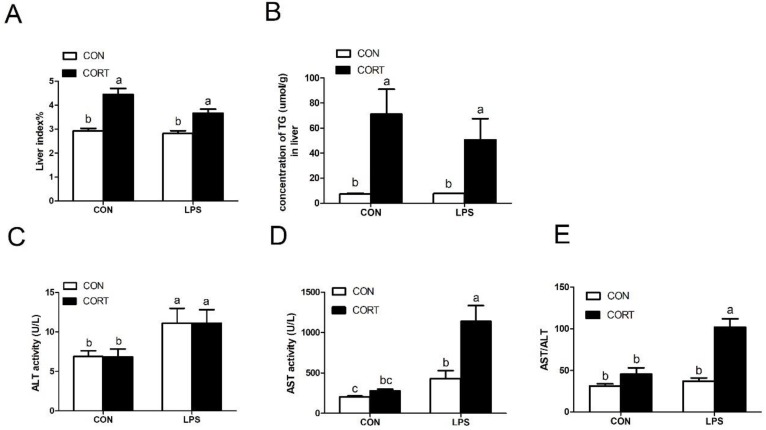
Level of liver index and biochemical parameters. (**A**) Liver index; (**B**) Hepatic TG content; (**C**) plasma alanine aminotransferase (ALT) activity; (**D**) plasma aspartate aminotransferase (AST) activity; (**E**) AST/ALT ratio. C-CON, control group; C-CORT, corticosterone-treated group; L-CON, LPS-treated group; L-CORT, LPS- and corticosterone-treated group. Data are presented as means ± SEM (n = 8). ^a–c^ Means with different small letter superscripts are significantly different (*p* < 0.05).

As shown in Figure 3, CORT challenge significantly increased the liver index and hepatic TG content (*p* < 0.05), but no differences were observed following LPS administration. Moreover, compared with the CON group, LPS injection significantly enhanced plasma ALT and AST activities (*p* < 0.05), but did not change the AST/ALT ratio. CORT administration had no effect on plasma activity or ratio. Interestingly, when combined with CORT, LPS exacerbated AST activity and altered the AST/ALT ratio when compared to the LPS group (*p* < 0.05).

### 3.5. Hepatic Expression of Genes Encoding Acute-Phase Proteins

**Figure 4:**
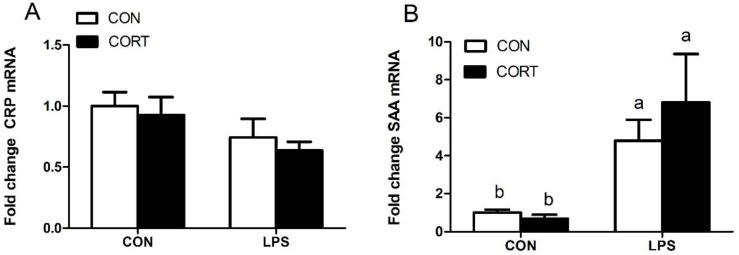
Expression of hepatic genes related to acute-phase proteins. (**A**) Hepatic expression of CRP mRNA. (**B**) Hepatic expression of SAA mRNA; C-CON, control group; C-CORT, corticosterone-treated group; L-CON, LPS-treated group; L-CORT, LPS- and corticosterone-treated group. Data were normalized to the expression of the 18S rRNA gene and are presented as means ± SEM (n = 8). ^a–c^ Means with different small letter superscripts are significantly different (*p* < 0.05).

As shown in Figure 4, LPS, but not CORT, significantly upregulated hepatic expression of *SAA* mRNA (*p* < 0.05). Hepatic expression of *CRP* mRNA did not change following treatment with either CORT or LPS.

### 3.6. Liver Expression of Genes Related to Inflammation

**Figure 5:**
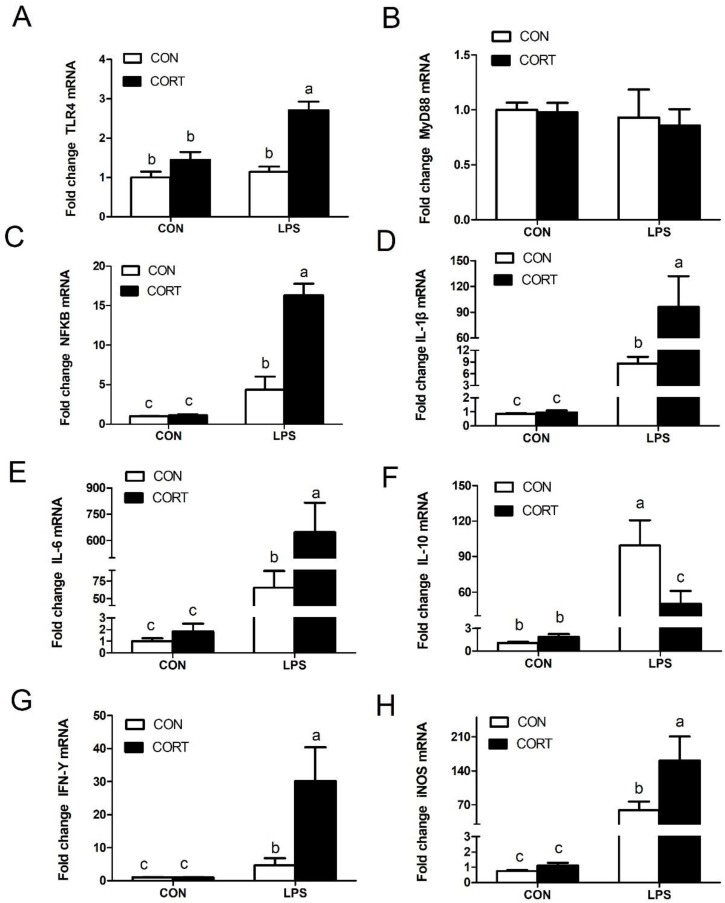
Expression of hepatic genes related to inflammation. (**A**) Hepatic TLR4 mRNA expression. (**B**) Hepatic MyD88 mRNA expression. (**C**) Hepatic NFκB mRNA expression. (**D**) Hepatic IL-1β mRNA expression. (**E**) Hepatic IL-6 mRNA expression. (**F**) Hepatic *IL-10* mRNA expression. (**G**) Hepatic IFN-γ mRNA expression; (**H**) Hepatic iNOS mRNA expression. C-CON, control group; C-CORT, corticosterone-treated group; L-CON, LPS-treated group; L-CORT, LPS- and corticosterone-treated group; Data were normalized to the expression of the 18S rRNA gene and are presented as means ± SEM (n = 8). ^a–c^ Means with different small letter superscripts are significantly different (*p* < 0.05).

Compared to the CON group, the CORT challenge was not able to induce an inflammatory response. However, LPS treatment greatly upregulated liver expression of downstream genes to the LPS-TLR4-NF-κB pathway. It also activated gene expression of hepatic *iNOS, IFN-γ, IL-1β*, and *IL-6* (*p* < 0.05; Figure 5). In particular, LPS injection exacerbated the expression of NF-κB, IL-1β, IL-6, IFN-γ, and iNOS in the liver when combined with CORT injections (*p* < 0.05). With respect to the anti-inflammatory cytokine, *IL-10*, expression of its mRNA was significantly upregulated by CORT or LPS injection, but was significantly downregulated by co-administration of CORT and LPS (*p* < 0.05).

## 4. Discussion

Fatty liver disease not only poses a considerable threat to human health, but also increases the risk of infection in the poultry industry. Broadly similar findings were observed in broilers following chronic CORT treatment, showing reduced protein synthesis and promoted protein degradation. Together, these cause loss of body weight [8,24]. In this study, body weight of CORT-treated broilers was lower than in the control under the same dietary formulations (Figure 1), in line with previous studies. Hepatocellular steatosis is a characteristic of NAFLD. More than 5% of hepatocyte steatosis is necessary for the diagnosis of NAFLD [25,26]. In addition, it has been reported that exacerbations of steatosis are positively correlated with lobular inflammation, zone-3 fibrosis, and definite steatohepatitis [25]. The gross appearance of the liver and HE staining of liver sections in broilers with CORT injection-induced fatty liver disease in our results support these findings (Figure 2). In rats, chronic treatment with dexamethasone (DEX), a synthetic GC, led to an increase in TG levels both in the blood and in the liver [27]. Similarly, in chickens, CORT induced a significant increase in plasma and hepatic TG content [8,28]. Our results were consistent with these studies, showing that CORT treatment significantly increased plasma levels of TG, CHOL, HDLC, and LDLC. These results also confirmed that the fatty liver model was successfully induced in the broilers, as in previous reports [8,10].

Many studies have shown that LPS can induce an inflammatory response in broilers [29]. The activities of AST and ALT in the plasma are usually used clinically as specific indicators of liver injury [30]. Our results are consistent with those of Chen et al. [31], who reported that LPS caused significant increases in levels of AST and ALT, as well as in hepatocyte necrosis and the accompanying inflammatory cell infiltration. Our results indicate that broilers treated with combined LPS and CORT showed higher plasma levels of AST and the AST/ALT ratio than following LPS or CORT treatment alone (Figure 3). In addition, histological analysis confirmed hepatic pathological changes. These results indicate that the inflammatory response could be exacerbated in broilers with fatty liver disease.

Two short acute-phase proteins of CRP and SAA expressed by hepatocyte act as a systemic response to local inflammation [32]. Our results show that LPS treatment significantly upregulated liver gene expression of SAA but not of CRP (Figure 4). This indicates that SAA was a sensitive indicator of gram-negative bacterial infection in this study.

As an important immune organ, the liver is a target for LPS, and is primarily affected by bacterial LPS [33,34]. In this study, results show that liver gene expression of proinflammatory cytokines was enhanced by LPS administration. Consistent with these results, Yang et al. [33] found that liver gene expression of *IL-6*, *TNF α*, and *iNOS* was considerably higher in the LPS-treated group than in the unchallenged broilers. Notably, although CORT treatment alone did not induce inflammatory gene expression, it could exacerbate LPS-induced mRNA expression of *NF-κB* and its downstream proinflammatory cytokines, such as *TNF-α* and *iNOS*, in broilers. A crosstalk occurs between steatosis and inflammation, influencing the hepatic immune system and the progression of NFALD [35]. Bertola et al. [36] found that livers of obese patients showed slight inflammation and were more sensitive to activators of the TLR pathway, even when histological abnormalities were absent. The outcome was the development of liver disease. However, the change in inflammatory gene expression was not consistent with the findings of Forn-Cuni and coworkers [37], who reported no significant change in inflammatory gene expression, when compared to the expression of LPS-stimulated and non-stimulated livers in obese zebrafish. These findings indicate that a fatty liver enhances the response to acute inflammatory stimuli and is dependent on health status of the animals, dose of the LPS injection, and the animal species.

## 5. Conclusions

Broilers with fatty livers are more susceptible to LPS stimulation, showing activation of the inflammatory response and more severe damage to the liver. LPS stimulation in broilers with fatty livers represent a second assault on the already damaged liver. Our findings extend the knowledge on the potential threat of pathogenic infections in the presence of fatty liver disease in poultry.

## Figures and Tables

**Table 1:** The composition of a chicken’s diet.

Ingredient %	Content
Corn	58.80
Soybean meal	30.50
Corn gluten meal	5.00
soybean oil	1.42
Calcium carbonate powder	1.34
Calcium hydrogen phosphate	1.33
Methionine	0.18
65% Lysine	0.45
93% Threonine	0.05
Sodium chloride	0.30
50% Choline chloride	0.30
Chlortetracycline	0.10
Trace elements	0.20
Vitamins	0.03
**Calculated Chemical Composition**	
Metabolizable energy (MJ/kg)	12.34
Crude protein (g/kg)	221.53
Crude fat (g/kg)	43.58
Crude fiber (g/kg)	27.90
Calcium (g/kg)	8.90
Available P (g/kg)	3.50
Lysine (g/kg)	12.91
Methionine (g/kg)	5.50
Methionine + Cysteine (g/kg)	9.17

**Table 2:** Nucleotide sequences of specific primers.

Target Genes	Genbank Accession	Primer Sequences (5′-3′)	PCR Products (bp)
*TLR4*	NM_001030693	F: AAACACCACCCTGGACTTGGAC	188
		R: GTGCTGGAGTGAATTGGCAGTTA
*MyD88*	NM_001030962	F: CCTGGAAAGTGATGAATGTGA	165
		R: TTGGTGCAAGGATTGGTGTA
*NF-κB*	NM_205134.1	F: TCAACGCAGGACCTAAAGACAT	162
		R: GCAGATAGCCAAGTTCAGGATG
*INF-γ*	X99774.1	F: ACACTGACAAGTCAAAGCCGCACA	129
		R: AGTCGTTCATCGGGAGCTTGGC
*iNOS*	D85422	F: CCTGGAGGTCCTGGAAGAGT	185
		R: CCTGGGTTTCAGAAGTGGC
*IL-6*	AJ309541.1	F: CGTGTGCGAGAACAGCATGGAGA	130
		R: TCAGGCATTTCTCCTCGTCGAAGC
*IL-1β*	Y15006	F: CATTCAGCGTCCAGGTA	91
		R: TCTCGGCAGTTTCTTTT
*IL-10*	NM_001004414.2	F: AGCAGATCAAGGAGACGTTC	103
		R: ATCAGCAGGTACTCCTCGAT
*CRP*	DQ374639.1	F: TGTCACGGCCCAGGAAGA	175
		R: TTTGGTGGCGTAGGAGAAGA
*SAA*	GU929209.1	F: AGCGGTATCAAGTTTGTCAGG	129
		R: TCCGAGCATCGTAATT
*18S*	>AF173612.1	F: GAAACGGCTACCACATCC	168
		R: CACCAGACTTGCCCTCCA	

*TLR4*, toll-like receptor4; *MyD88*, myeloid differentiation factor88; *NFκB*, nuclear factor kappa-light-chain-enhancer of activated B cells; *IFN-γ*, interferon γ; *iNOS*, inducible nitric oxide synthase; *IL-6*, *IL-1β*, and *IL-10*, interleukin 6, 1β, and 10, respectively; *CRP*, C-reactive protein; *SAA*, serum amyloid A.

**Table 3:** Effect of CORT and LPS on plasma biochemical parameters.

Parameters	CON	CORT	LPS	LPS&CORT
TG (mmol/l)	0.40 ± 0.03 ^b^	0.70 ± 0.10 ^a^	0.27 ± 0.02 ^c^	0.64 ± 0.14 ^a,b^
TCHOL (mmol/l)	2.67 ± 0.22 ^c^	5.66 ± 0.53 ^a^	2.24 ± 0.18 ^c^	4.23 ± 0.32 ^b^
HDL (mmol/l)	1.48 ± 0.12 ^c^	3.25 ± 0.27 ^a^	1.14 ± 0.12 ^c^	2.22 ± 0.17 ^b^
LDL (mmol/l)	0.28 ± 0.02 ^b^	0.53 ± 0.08 ^a^	0.39 ± 0.03 ^a,b^	0.44 ± 0.06 ^a^
GLU (mmol/L)	14.80 ± 0.31	14.80 ± 0.48	14.40 ± 0.64	15.81 ± 0.67

CON, control group; CORT, corticosterone-treated group; LPS, LPS-treated group; LPS&CORT, LPS- and corticosterone-treated group. TG, triglycerides; TCHOL, total cholesterol; HDL, high density lipoprotein cholesterol; LDL, low density lipoprotein cholesterol; GLU, glucose. Data are presented as means ± SEM (n = 8). ^a–c^ Means with different small letter superscripts within a row are significantly different (*p* < 0.05).
